# Generation of stem cell-derived β-cells from patients with type 1 diabetes

**DOI:** 10.1038/ncomms11463

**Published:** 2016-05-10

**Authors:** Jeffrey R. Millman, Chunhui Xie, Alana Van Dervort, Mads Gürtler, Felicia W. Pagliuca, Douglas A. Melton

**Affiliations:** 1 Division of Endocrinology, Metabolism and Lipid Research, Washington University School of Medicine, Campus Box 8127, 660 South Euclid Avenue, St Louis, Missouri 63110, USA; 2 Department of Biomedical Engineering, Washington University in St Louis, 1 Brookings Drive, St Louis, Missouri 63130, USA; 3 Department of Stem Cell and Regenerative Biology, Harvard Stem Cell Institute, Harvard University 7 Divinity Avenue, Cambridge, Massachusetts 02138, USA; 4 Case Western Reserve University School of Medicine, 2109 Adelbert Road, Cleveland, Ohio 44106, USA; 5 Semma Therapeutics, Inc. 450 Kendall Street, Suite 2B Cambridge, Massachusetts 02142, USA

## Abstract

We recently reported the scalable *in vitro* production of functional stem cell-derived β-cells (SC-β cells). Here we extend this approach to generate the first SC-β cells from type 1 diabetic patients (T1D). β-cells are destroyed during T1D disease progression, making it difficult to extensively study them in the past. These T1D SC-β cells express β-cell markers, respond to glucose both *in vitro* and *in vivo*, prevent alloxan-induced diabetes in mice and respond to anti-diabetic drugs. Furthermore, we use an *in vitro* disease model to demonstrate the cells respond to different forms of β-cell stress. Using these assays, we find no major differences in T1D SC-β cells compared with SC-β cells derived from non-diabetic patients. These results show that T1D SC-β cells could potentially be used for the treatment of diabetes, drug screening and the study of β-cell biology.

## Abstract (web summary)

Pancreatic β cells can be generated from pluripotent stem cells. Here, the authors show that human induced pluripotent stem cells from patients with type 1 diabetes can be differentiated into β-like cells that have no detectable differences compared with cells from non-diabetic individuals.

## Graphical abstract

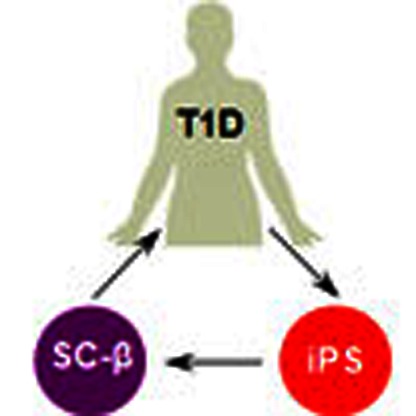

Patient-derived human induced pluripotent stem cells (hiPSCs), differentiated to disease-relevant cells, are becoming quite important due to their potential for cell replacement therapy and drug screening, as well as improving our understanding of the pathophysiology of disease. Type 1 diabetes (T1D) occurs by autoimmune-mediated destruction of pancreatic β-cells, and genome-wide association studies have revealed that most genetic loci associated with T1D are affiliated with the immune system. However, several loci and related networks are expressed in the β-cells or are otherwise non-immune^1^,^2^,^3^. The role intrinsic defects in β-cells from patients, such as reduced mass and function or susceptibility and response to stress, may play in initiating the disease remains unclear^1^,^2^,^3^,^4^,^5^,^6^,^7^. Furthermore, what T1D patient-specific barriers, if there are any, may impede the use of autologous hiPSC technology for cell replacement therapy are unknown. As β-cells are destroyed during disease progression, procurement of β-cells from T1D patients that have not undergone disease-related environmental stress for study has not been possible.

Transplantation of exogenous β-cells to replace dead or dysfunctional endogenous β-cells is a potential strategy for controlling blood glucose levels in diabetic patients. Allogeneic transplantation of cadaveric islets has already been performed on patients with positive clinical results, but this approach suffers from a limited islet supply and the requirement that patients remain on immunosupressants^8^. Human pluripotent stem cells^9^, including both human embryonic stem cells (hESCs)^10^,^11^,^12^,^13^ and hiPSC^13^,^14^,^15^,^16^, provide the basis for potentially unlimited numbers of replacement cells. Several groups have detailed the generation of early and intermediate cell types from human pluripotent stem cells, such as definitive endoderm and pancreatic progenitors^10^,^11^,^12^,^13^. Cells that express low levels of insulin, but few other β-cell markers, have been generated from T1D hiPSC previously. However, these cells have been of limited utility, as they do not resemble *bona fide* β-cells, lack function *in vitro* and *in vivo*, mis-express β-cell and other islet genes, and lack correct granular ultrastructure^15^,^16^. In recent times, we reported the generation of SC-β cells from both hESC- and hiPSC-derived from non-diabetic (ND) donors that have many of the defining features of β-cells, including its function both *in vitro* and *in vivo*, β-cell marker expression and the ability to control glucose levels in diabetic mice^17^,^18^. Other groups have reported similar protocols and findings^19^,^20^.

Here we use this novel technology to generate SC-β cells from T1D hiPSC for the first time. These cells, referred to as T1D SC-β cells, express markers found in β-cells, are functionally indistinguishable from ND SC-β cells *in vitro* and *in vivo*, and prevent alloxan-induced diabetes in mice. Insulin secretion is increased in these T1D SC-β cells in response to several categories of anti-diabetic drugs. Furthermore, we show that these cells respond to different types of chemically induced stress, develop an *in vitro* disease model of T1D SC-β cell stress and demonstrate a partial rescue of this stress phenotype with treatment of a small molecule (an Alk5 inhibitor). T1D SC-β cells can be used to better study diabetes and as a potential autologous source for cell replacement therapy.

## Results

### Derivation and *in vitro* assessment of T1D SC-β cells

**Figure 1:**
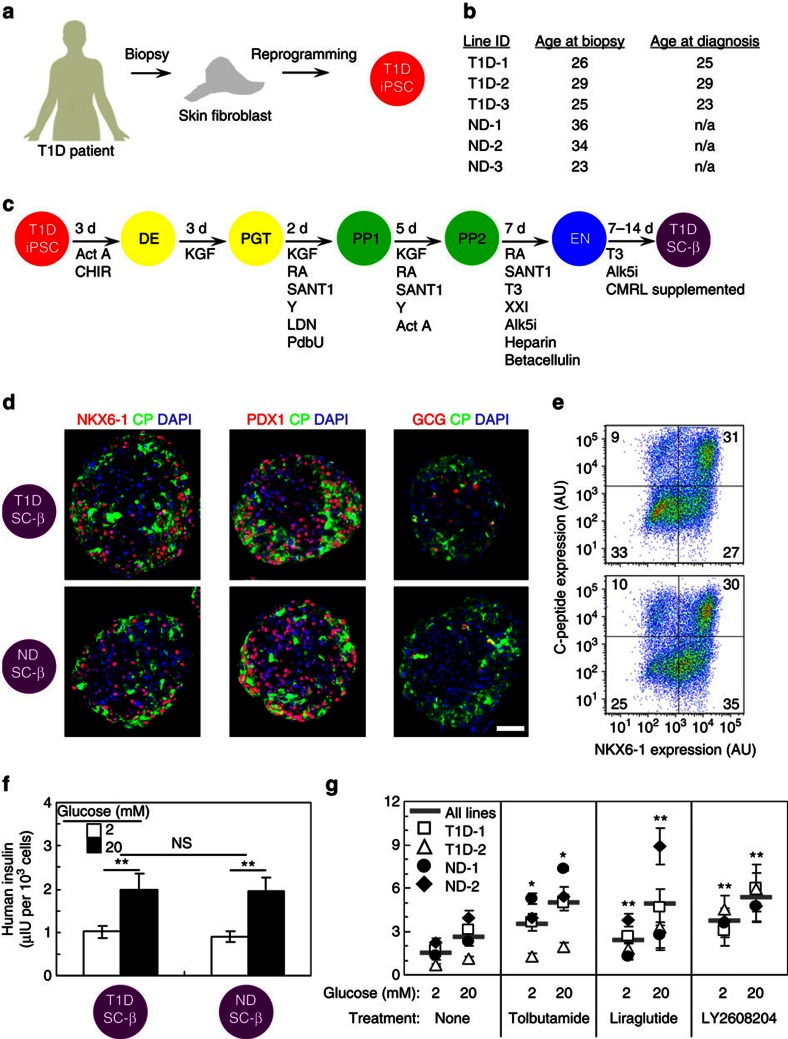
T1D SC-β cells express β-cell markers and secrete insulin in response to high glucose and anti-diabetic drug treatment *in vitro*. (**a**) Schematic summarizing derivation of hiPSC from T1D patients. (**b**) Table of cell lines used in study, showing donor age at the time of skin biopsy procurement and age of diagnosis of T1D, if applicable. (**c**) Schematic summarizing differentiation protocol used to produce SC-β cells. (**d**) Representative immunostaining of T1D and ND SC-β cells stained for NKX6-1, PDX1, glucagon (GCG; red) with C-peptide (CP; green) and 4,6-diamidino-2-phenylindole (DAPI) (blue). Scale bar, 100 μm. (**e**) Representative flow cytometry plot of dispersed clusters stained for C-peptide and NKX6-1. AU, arbitrary units. (**f**) Average ELISA measurements of secreted human insulin at 2 and 20 mM glucose. *n*=9 and 9 SC-β cell batches, each consisting of 3 T1D donors and 3 ND donor. **P<0.01 comparing 20 and 2 mM glucose treatments (two-sided paired *t*-test). (**g**) ELISA measurements of secreted human insulin at 2 and 20 mM glucose for a subset of cell lines treated with the anti-diabetic drugs Tolbutamide (sulfonylurea), Liraglutide (GLP-1 R agonist) and LY2608204 (GCK activator). *n*=4 measurements for each line. *P<0.05 and **P<0.01 comparing drug treatment with no treatment at the same glucose concentration (two-sided unpaired *t*-test). Data shown as mean±s.e.m. Samples taken after 10–17 days in Stage 6. Act A, activin A; Alk5i, Alk5 receptor inhibitor II; CHIR, CHIR9901; KGF, keratinocyte growth factor; LDN, LDN193189; PdbU, phorbol 12,13-dibutyrate; RA, retinoic acid; T3, triiodothyronine; Y, Y27632.

To generate T1D and ND SC-β cells, we derived and characterized hiPSC from skin fibroblasts of patient donors (Fig. 1a,b). As described previously^15^, we found both T1D and ND hiPSC to express pluripotent stem cell markers, differentiate to express markers of all three germ layers and, after undergoing planar differentiation to pancreatic progenitors, produce PDX1+/NKX6-1+ cells that can be transplanted into mice to spontaneously generate glucose-responsive cells *in vivo* (Supplementary Figs 1 and 2).

After T1D and ND hiPSC derivation and characterization, we adapted the cells to suspension culture to undergo differentiation to produce SC-β cells (Fig. 1c)^17^. We generated numerous batches of cells from all six cell lines. A batch of cells is a single flask of cells that has undergone the entire differentiation protocol and is considered a single biological replicate. Both T1D and ND hiPSC are capable of differentiating to SC-β cells that co-express C-peptide+/NKX6-1+ and C-peptide+/PDX1+, with few cells expressing the α-cell hormone glucagon (Fig. 1d). When quantified by flow cytometry, we found on average 24±2% and 27±2% of cells co-expressed C-peptide+/NKX6-1+ for T1D and ND cells, respectively (Fig. 1e), similar to what we previously reported with hESCs and ND hiPSCs^17^. Furthermore, when analysed with electron microscopy, both T1D and ND SC-β cells contained developing and mature insulin granules that are similar to β-cell granules (Supplementary Fig. 3)^17^.

### T1D SC-β cells function and respond to anti-diabetic drugs

We next tested the cells with an *in vitro* glucose-stimulated insulin secretion assay, to assess their function. We found that both T1D and ND SC-β cells can respond to sequential glucose challenges (Supplementary Fig. 4). On average for 18 biological batches (9 for T1D and 9 for ND), T1D and ND SC-β cells secrete 2.0±0.4 and 1.9±0.3 μIU of human insulin per 10^3^ cells in response to 20 mM glucose and have stimulation indexes (ratio of insulin released at 20–2 mM glucose) of 1.9 and 2.2, respectively (Fig. 1f). On average, T1D and ND cells responded to 88% and 78% of the challenges, respectively. Insulin content was similar between the two groups, 210±40 μIU per 10^3^ cells and 220±20 μIU per 10^3^ for T1D (*n*=3 batches, 1 from each donor) and ND (*n*=3 batches, 1 from each donor), respectively. Overall, these T1D SC-β cells function similar to ND SC-β cells generated in this study and to our previous report of SC-β cells and adult primary islets^17^.

As a proof-of-concept for their possible use in drug screening, a subset of T1D and ND SC-β cell lines were treated with three anti-diabetic compounds that affect insulin secretion by different mechanisms: Sulfonylurea (tolbutamide), GLP-1R agonist (Liraglutide) and GCK activator (LY2608204) (Fig. 1g). Treatment with each of the three compounds increases insulin release at both low- and high-glucose challenges compared with control by a factor of 2.0 on average.

### T1D SC-β cells function after transplantation

**Figure 2:**
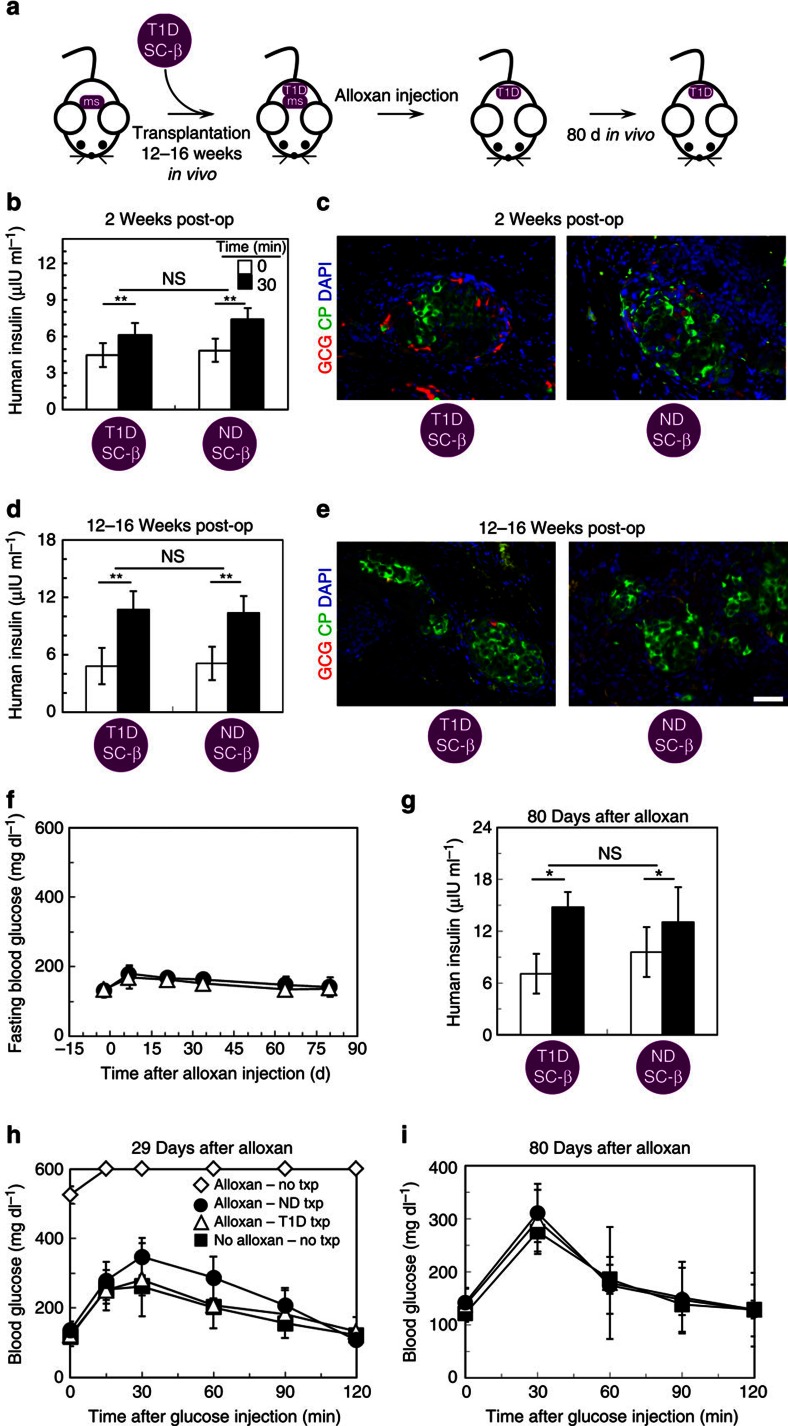
T1D SC-β cells function rapidly and persist several months *in vivo* after transplantation. (**a**) Schematic summarizing *in vivo* transplantation experiments. ms, endogenous mouse β cells. T1D, T1D SC-β cells. (**b**) Average ELISA measurements of serum human insulin before (0 min) and 30 min after a glucose injection for mice that received T1D and ND SC-β cells 2 week prior. *n*=32 and 48 for T1D and ND SC-β cells, respectively. (**c**) Representative immunostaining of grafts 2 weeks after transplantation stained with glucagon (GCG; red), C-peptide (green) and 4,6-diamidino-2-phenylindole (DAPI) (blue). (**d**) Average ELISA measurements of serum human insulin before (0 min) and 30 min after a glucose injection for mice that received T1D and ND SC-β cells 12–16 weeks prior. *n*=15 and 18 for T1D and ND SC-β cells, respectively. (**e**) Representative immunostaining of grafts 12–16 weeks after transplantation. (**f**) Fasting blood glucose measurements for a subset of mice that received an alloxan injection to destroy endogenouse mouse β cells. Mice that were transplanted with ND SC-β cells (closed circle; *n*=4) and with T1D SC-β cells (open triangle; *n*=4) are shown. (**g**) Average ELISA measurements of serum human insulin before (0 min) and 30 min after a glucose injection for mice that were injected with alloxan 80 days prior. *n*=4 and 4 for T1D and ND SC-β cells, respectively. (**h**) Temporal blood glucose measurements after a glucose injection 29 days after alloxan treatment. Shown are mice that had not received a transplantation (open diamond; *n*=2), were transplanted with ND SC-β cells (closed circle; *n*=4), were transplanted with T1D SC-β cells (open triangle; *n*=4), or did not receive alloxan treatment and did not receive a transplantation (closed square; *n*=5) are shown. (**i**) Temporal blood glucose measurements after a glucose injection 80 days after alloxan treatment. Mice that were transplanted with ND SC-β cells (closed circle; *n*=4), were transplanted with T1D SC-β cells (open triangle; *n*=4), or did not receive alloxan treatment and did not receive a transplantation (closed square; *n*=4) are shown. Mice were transplanted with cells 10–17 days in Stage 6. Scale bar, 100 μm. *P<0.05 and **P<0.01 (two-sided paired t-test). Data shown as mean±s.e.m.

To evaluate their potential use in cell replacement therapy and *in vivo* physiological tests and further confirm their identity as SC-β cells, T1D and ND SC-β cells were transplanted underneath the kidney capsule of ND immunocompromised mice (Fig. 2a). After 2 weeks, graft function was evaluated by measuring serum human insulin before and 30 min after an injection of glucose (Fig. 2b and Supplementary Table 1). At this early time point, human insulin is detected and the grafts were glucose responsive in most, but not all, mice. Overall, 81% (26/32) and 77% (37/48) secreted more human insulin after glucose injection, for T1D and ND SC-β cells, respectively. The ratio of insulin secretion after glucose challenge compared with before challenge averaged 1.4 and 1.5, for T1D and ND SC-β cells, respectively. Again, no major differences between these T1D and ND SC-β cells were observed^17^. Immunostaining of recovered grafts revealed clusters of C-peptide+ cells with some glucagon+ cells (Fig. 2c).

The presence of both T1D and ND SC-β cells were observed for several months after transplantation, with the grafts continuing to respond to glucose injections and high amounts of human insulin being detected in the serum (Fig. 2d,e). After 3–4 months, a subset of mice were treated with alloxan to selectively kill mouse but not human β-cells^21^, to evaluate the ability of T1D and ND SC-β cells to maintain euglycemia (Fig. 2a,c). Destruction of endogenous mouse β-cells was confirmed by demonstrating the absence of serum mouse C-peptide. Mice that did not receive a SC-β cell graft but were treated with alloxan died within 29 days of alloxan treatment. Mice were observed for up to 80 days after alloxan treatment, for a total of 164–192 days since SC-β cell transplantation, with mice that received either T1D or ND SC-β cells maintaining on average <200 mg dl^−1^ fasting blood glucose (Fig. 2f), secreting human insulin in response to a glucose injection (Fig. 2g) and rapidly clearing glucose after a glucose injection (Fig. 2h,i). This data shows that grafts continue to function for >5 months *in vivo*.

### Development of a disease model of T1D SC-β cell stress

**Figure 3:**
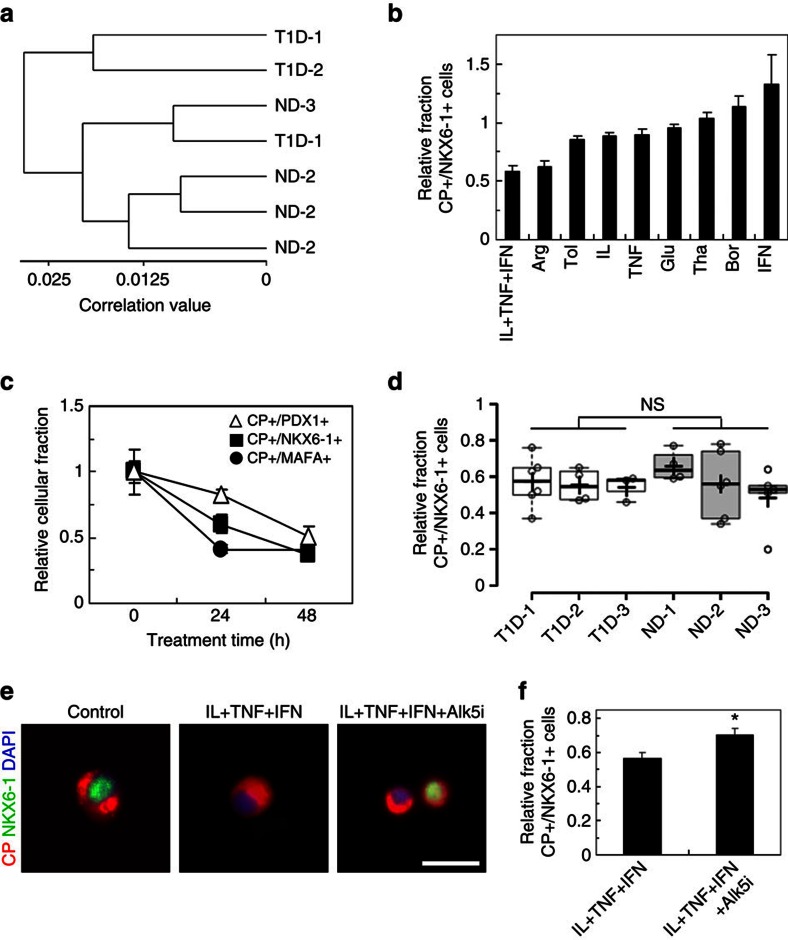
T1D and ND SC-β cells have similar gene expression and response to cytokine stress. (**a**) Hierarchical clustering based on global gene expression measured by microarray of a subset of T1D and ND SC-β cell lines sorted for INS and NKX6-1. Individual replicates shown. (**b**) The average relative fraction of T1D and ND SC-β cells immunostained for C-peptide (CP) and NKX6-1 treated 24 h with the indicated stressor normalized by untreated cells. *n*=3 T1D and 4 ND SC-β cells batches (7 batches total). (**c**) The average relative fraction of cells immunostained for C-peptide and PDX1, NKX6-1 or MAFA treated up to 48 h with interleukin (IL)-1β, tumour necrosis factor (TNF)-α and interferon (IFN)-γ *n*=3 T1D and 4 ND SC-β cells batches (7 batches total). (**d**) Box and whiskers plot comparing of the relative fraction of C-peptide+/NKX6-1+ cells for T1D-1 (*n*=6), T1D-2 (*n*=4), T1D-3 (*n*=3), ND-1 (*n*=4), ND-2 (*n*=6) and ND-3 (*n*=5) after 24 h treatment with IL-1β, TNF-α and IFN-γ (28 batches total). The cross indicates the mean, the line the median and each circle is one biological replicate. (**e**) Representative images of immunostained cells not treated, treated with IL-1β, TNF-α and IFN-γ, or treated with IL-1β, TNF-α, IFN-γ and Alk5i for 24 h. Scale bar, 20 μm. (**f**) Average relative fraction of C-peptide+/NKX6-1+ cells treated with IL-1β, TNF-α and IFN-γ either without (left) or with (right) Alk5i for 24 h. *n*=10 T1D and 7 ND SC-β cells batches (17 batches total). Quantification of immunostained cells in this figure was performed with Cellomics ArrayScanVTI. *P<0.05 (two-sided paired *t*-test). Experiments were performed on and samples taken from cells after 10–17 days in Stage 6. Arg, arginine; Bor, bortezomib; Glu, glucose; IFN, interferon-γ; IL, interleukin-1β; Tha, Thapsagargin; TNF, tumour necrosis factor-α; Tol, tolbutamide. Data shown as mean±s.e.m.

The results summarized above support the conclusion that T1D SC-β cell function is similar to that of ND SC-β cells both *in vitro* and *in vivo*. To further compare the two types of SC-β cells, we focused on a subset of cell lines and employed a method we recently reported to analyse global gene expression from fixed, immunostained and sorted cell populations. (Fig. 3a, Supplementary Fig. 5 and Supplementary Data 1)^17^,^22^. There is little, if any, major difference between these T1D and ND SC-β cells as indicated by the clustering of the samples. Furthermore, these cells clustered with our previously reported hESC SC-β cells and adult cadaveric β-cells, but are distinct from fetal β-cells and from T1D and ND hiPSC that had undergone a control planar protocol that produces dysfunctional insulin-expressing cells.

Increased cellular susceptibility to exogenous stress has been reported in patient-derived hiPSC from some diseases, including ALS and Wolfram syndrome^23^,^24^. Similarly, β-cells are known to be susceptible to stress, which may play a role in initiating diabetes^3^,^4^,^5^,^25^,^26^. To study this in T1D, the *in vitro*-derived SC-β cells were acutely subjected to several chemical stressors and the persistence of SC-β cells was evaluated through assessment of the co-expression of β-cell markers C-peptide+/NKX6-1+ (Fig. 3b and Supplementary Fig. 6). Many of the stress treatments reduced the fraction of cells co-expressing C-peptide+/NKX6-1+, with the cytokine stress cocktail (a combination of interleukin-1β (IL), tumour necrosis factor-α (TNF) and interferon-γ (IFN)) resulting in the largest reduction.

Given the role that cytokines and inflammation are thought to play in T1D^25^, we chose to further investigate the effects of interleukin-1β, tumour necrosis factor-α and interferon-γ on T1D and ND SC-β cells. Cytokine treatment results in the rapid loss of not only C-peptide+ cells that co-express NKX6-1+ but also cells that co-express MAFA+ (Supplementary Fig. 7) and PDX1+. The reduction of MAFA and NKX6-1 precedes PDX1, as has been reported for primary human β-cells^27^ (Fig. 3c). We hypothesized that the T1D SC-β cells would be more sensitive to cytokine-induced stress than ND SC-β cells, as has been reported in other patient hiPSC disease systems^23^,^24^. However, no detectable difference in the T1D and ND SC-β response to cytokine-induced stress was observed (Fig. 3d).

The stress conditions identified in Fig. 3 provide the potential basis for a human disease model drug-discovery platform. To demonstrate its utility for drug screening, cells in this cytokine stress assay were treated with Alk5 inhibitor type II (Alk5i), a compound that was previously identified to prevent stress-induced phenotypic loss in mouse β-cells^28^. With human SC-β cells, treatment with Alk5i partially blocks loss of C-peptide+/NKX6-1+ cells, improving retention by 32% (Fig. 3e,f).

## Discussion

In summary, we demonstrate that functional SC-β cells can be generated from hiPSC derived from T1D patients *in vitro* (Fig. 1a–c). These cells are very similar to those we previously reported for hESC and ND hiPSC SC-β cell and are similar but not identical to adult β-cells^17^. T1D SC-β cells express markers found in β-cells, including NKX6-1 and PDX1, and have global gene expression patterns similar to adult β-cells (Figs 1d,e and 3a). T1D SC-β cells are functional *in vitro* and *in vivo*, responding to glucose challenges by increasing their human insulin release and controlling glucose levels in diabetic mice after transplantation (Fig. 2). After exposure to chemical-induced stress, T1D SC-β cells lose expression of β-cell markers (Fig. 3b,e). These cells also respond to cytokine stress, which can be partially prevented by treatment with Alk5i (Fig. 3b–f)^28^. Finally, these cells also respond to known anti-diabetic drugs that cause increased insulin secretion (Fig. 1g).

These experimental approaches could directly be used for discovery of novel compounds that increase function and prevent stress and death in β-cells. The usefulness of these cells could be further enhanced by a combination with recently reported genetic modification strategies, for example, by the use of TALEN or CRISPR-Cas9 to introduce or correct known diabetes-associated mutations^29^,^30^ or engineering reporter constructs to improve functional readout^31^.

Overall, we do not observe major differences in SC-β cells derived from T1D and ND patients with the assays and cell lines used in this report. It is important to note that this does not mean differences do not exist in T1D β-cells compared with ND β-cells. One possibility is that defects or differences would appear after extended culture or many years after transplantation. Diabetes generally develops over a long period, and we did not test for the effects of ageing. In addition, investigating how T1D SC-β cells interact with the immune system would be quite informative. Indeed, the gene with the largest differential expression in T1D and ND SC-β cells is TAP1, a peptide transporter associated with the major histocompatibility complex that has been implicated in with T1D^32^. In addition, it is important to note that the three T1D donors used in this study do not represent the whole T1D population; differences among patients are likely to exist.

The lack of observable gross deficiencies in T1D SC-β cells combined with their functional phenotype indicates the potential utility of these autologous cells in cell replacement therapy for diabetes for at least a proportion of the T1D population. We caution that additional studies need to be conducted with a larger number of patient-specific cell lines before clinical use. In combination with a strategy to prevent autoimmune destruction^33^, these cells may provide an alternative to current approaches in transplantation that have to address the additional hurdle of allo- or xenogeneic rejection.

## Methods

### Cell line derivation and characterization

ND-1 and ND-2 were previously published^17^. Other hiPSC lines were derived from fibroblasts grown out from skin biopsies received from Columbia University using Sendai viral reprogramming on mouse fibroblasts (Life Technologies; A1378001). Cell lines were characterized by karyotype analysis, expression of pluripotency genes and differentiation genes in a spontaneous differentiation assay. G-banded karyotype analysis was performed by Cell Line Genetics (Madison, WI). In brief for the other assays, colonies were stained with antibodies for TRA-1-60 (Millipore; MAB4360), SSEA-3 (Millipore; MAB4303), SSEA-4 (Millipore; MAB4304), NANOG (Abcam; ab21624) and OCT4 (Abcam; ab19857). Colonies were also stained for alkaline phosphatase (Millipore; SCR004) and reverse transcriptase–PCR performed for DNMT3B, HTERT, NANOG, OCT4, REX1 and SOX2. Differentiation was assessed by a spontaneous embryoid body differentiation and assessment of markers for the three germ layers, using reverse transcriptase–PCR to look for NCAM, PAX6, AFP, GATA4, FLK1 and GATA2. RNA was extracted using RNeasy Mini-Kit (Qiagen; 74106) and complementary DNA synthesized using qScript cDNA SuperMix (Quanta; 101,414–106). The primer sequences used are (gene, forward primer, reverse primer, annealing temperature and cycle number): ACTIN, 5′-ggacttcgagcaagagatgg-3′, 5′-agcactgtgttggcgtacagnnealing-3′, 60 °C, 25; DNMT3B, 5′-ataagtcgaaggtgcgtcgt-3′, 5′-ggcaacatctgaagccattt-3′, 56 °C, 30; HTERT, 5′-tgtgcaccaacatctacaag-3′, 5′-gcgttcttggctttcaggat-3′, 57 °C, 33; NANOG, 5′-tccaacatcctgaacctcag-3′, 5′-gactggatgttctgggtctg-3′, 58 °C, 30; OCT4 5′-gtggaggaagctgacaacaa-3′, 5′-caggttttctttccctagct-3′, 56 °C, 30; REX1, 5′-tggacacgtctgtgctcttc-3′, 5′-gtcttggcgtcttctcgaac-3′, 60 °C, 30; SOX2, 5′-ttgtcggagacggagaagcg-3′, 5′-tgaccaccgaacccatggag-3′, 64 °C, 33; NCAM, 5′-atggaaactctattaaagtgaacctg-3′, 5′-tagacctcatactcagcattccagt-3′, 68 °C, 33; PAX6, 5′-tctaatcgaagggccaaatg-3′, 5′-tgtgagggctgtgtctgttc-3′, 57 °C, 35; AFP, 5′-agcttggtggtggatgaaac-3′, 5′-ccctcttcagcaaagcagac-3′, 58 °C, 30; GATA4, 5′-ctagaccgtgggttttgcat-3′, 5′-tgggttaagtgcccctgtag-3′, 61 °C, 30; FLK1, 5′-agtgatcggaaatgacactgga-3′, 5′-gcacaaagtgacacgttgagat-3′, 63 °C, 32 and GATA2, 5′-gcaacccctactatgccaacc-3′, 5′-cagtggcgtcttggagaag-3′, 58 °C, 35.

### Cell culture

After adapting the undifferentiated cells to feeder-free culture on Matrigel (BD Biosciences; 08774552) in mTeSR1 (StemCell Technologies Inc.; 05850), undifferentiated cells were adapted and maintained in mTeSR1 in 500-ml spinner flasks (Corning; 89,089–814) on a stir plate (Chemglass) rotating at 70 r.p.m. in a humidified 37 °C incubator set at 5% CO_2_. Undifferentiated cells were maintained by passaging clusters dispersed with Accutase (StemCell Technologies; 07920) and seeded at 0.5 × 10^6^ cells per ml in mTeSR1 supplemented with 10 μM Y27632 (Abcam; ab120129). Cultures were passaged every 72–96 h (ref. 17).

To initiate differentiation to SC-β cells, clusters were dispersed as above and seeded between 0.25 and 1 × 10^6^ cells per ml in mTeSR1 supplemented with 10 μM Y27632 to achieve cluster diameters of 150–220 μm after 48–72 h. Differentiation was initiated by changing the media to differentiation media and following feeding schedule seen in Supplementary Tables 2 and 3. S6 basal media used in stage 6 was made by supplementing CMRL 1,066 Supplemented (Mediatech; 99-603-CV) with 10% fetal bovine serum (HyClone; 16,777) and 1% Penicillin/Streptomycin. For this study, we had three batches of differentiation that failed to produce at least 40% PDX1+/NKX6-1+ by the end of stage 4 and were not included in the analysis. SC-β cell samples were acquired and experiments on SC-β cells were performed after 10–17 days in Stage 6.

In a subset of experiments, PDX1+/NKX6-1+ pancreatic progenitors and dysfunctional cells that express C-peptide were generated with a planar differentiation protocol for initial evaluation of hiPSC differentiation capacity. To initiate planar differentiation, undifferentiated cells were seeded at 5.3 × 10^6^ cells per cm^2^ on plates treated with growth factor-reduced Matrigel (BD Biosciences; 356,231) in mTeSR1 supplemented with 10 μM Y27632 and after 24 h the media were changed to differentiation media. To generate PDX1+/NKX6-1+ pancreatic progenitors, the media were changed as seen in Supplementary Table 4. To generate PH cells, the media were changed as seen in Supplementary Table 5.

### Immunohistochemistry

Cell clusters or excised kidneys were fixed with 4% paraformaldehyde (PFA; Electron Microscopy Science; 15,714) for 1 h at room temperature (RT) or overnight at 4 °C, respectively, before being paraffin embedded and sectioned at 10 μm. Paraffin was removed with Histoclear (Thermo Scientific; C78-2-G) and rehydrated, and antigens retrieved by 2 h 0.1 M EDTA (Ambion; AM9261) treatment in a pressure cooker (Proteogenix; 2,100 Retreiver). Samples were blocked with 0.1% Triton X-100 (VWR; EM-9400) and 5% donkey serum (Jackson Immunoresearch; 017-000-121) in PBS (staining solution) for 1 h at RT, incubated with primary antibodies diluted in staining solution overnight at 4 °C, washed for 5 min in staining solution, incubated covered with appropriate Alexa Fluor-488 or -594 secondary antibodies diluted 1:300 in staining solution 2 h at RT, washed for 5 min in staining solution, mounted with Vectashield (Vector Laboratories; H-1200) and covered with a coverslip. Images were taken with an Olympus IX51 Microscope. The primary antibodies used to assess differentiation throughout this study are given in Supplementary Table 6 (ref. 17).

### Flow cytometry

Clusters were dispersed by treatment with TrypLE Express (Life Technologies; 12,604,013) at 37 °C, fixed with 30 min treatment with 4% PFA on ice, blocked by 30 min incubation in staining buffer, stained with primary antibodies diluted in staining buffer overnight at 4 °C, washed twice with staining buffer, stained with appropriate AlexaFluor-488 and -647 secondary antibody diluted 1:300 in staining buffer and washed twice with staining buffer. Stained cells were measured using an LSR II flow cytometer (BD Biosciences) and analysed using FlowJo^17^.

### Electron microscopy

Cellular ultrastructure was assessed with electron microscopy^17^. Clusters were fixed with 1.25% PFA, 2.5 glutaraldehyde and 0.03% picric acid in pH 7.4, 0.1 M sodium cacodylate for 2 h at RT, washed in 0.1 M cacodylate buffer, incubated at least 2 h at RT in 1% osmium tetroxide and 1.5% potassium ferrocyanide (OsO_4_/KFeCN_6_), washed in 0.1 M cacodylate buffer, incubated for 1 h in OsO_4_/KFeCN_6_, washed thrice with water, stained for 1 h in 1% aqueous uranyl acetate, washed twice in water and dehydrated with alcohol. After a 1-h incubation in propyleneoxide, samples were infiltrated overnight in 1:1 propyleneoxide and TAAB Epon (Marivac Canada), embedded, sectioned (60 nm thickness), placed onto copper grids, stained with 0.2% lead citrate and analysed using a JEOL 1200EX transmission electron microscope. Assessment of ultrastructure was performed by a blinded observer.

### *In vitro* glucose-stimulated insulin secretion

*In vitro* function was assessed by measuring *in vitro* glucose-stimulated insulin secretion^17^. Clusters were washed twice in Krebs buffer (krb), preincubated for 2 h in krb containing 2 mM glucose (low glucose) at 37  °C and washed once. Clusters were then challenged with three sequential treatments of alternating low-glucose krb and high-glucose krb containing 20 mM glucose (sx total glucose treatments), followed by treatment with low-glucose krb containing 30 mM KCl. Each treatment lasted 30 min, after which 100 μl of supernatant was collected and human insulin quantified using the Human Ultrasensitive Insulin ELISA (ALPCO Diagnostics; 80-INSHUU-E01.1. Human insulin measurements were normalized by viable cell counts that were acquired by dispersing clusters with TrypLE Expression and counted using a ViCell (Beckman Coulter). The formulation for krb buffer is seen in Supplementary Table 7. In a subset of experiments, cells were pre-cultured with an anti-diabetic drug for 24 h in stage 6 media and that same anti-diabetic drug was also included in the krb buffer: Tolbutamide (100 μM; Sigma; T0891), LY2608204 (1 μM; Selleck; S2155) and Liraglutide (1 μM; Bachem; H-6724.0001).

### Kidney capsule transplantation

All performed animal work was done in accordance to Harvard University International Animal Care and Use Committee regulations. The number of mice chosen was sufficient to statistically show function previously^17^. No randomization was used. All procedures were done by blinded individuals. ND male immunodeficient SCID/Beige mice (Taconic) aged 8–10 weeks were anaesthetized with 250 mg kg^−1^ avertin, an incision made to expose the kidney, a catheter needle inserted underneath the capsule and 5 × 10^6^ cells injected. Cells generated with planar protocols were aggregated overnight to form clusters before injection. After the incision site was closed, mice were given 5 mg kg^−1^ carprofen and another dose 24 h later. Mice were monitored twice a week after transplantation.

Two weeks after transplantation, the function of transplanted cells was assessed by performing *in vivo* glucose-stimulated insulin secretion. Mice were fasted 16 h and the blood collected before and 30 min after an intraperitoneal injection of 2 g kg^−1^ glucose by facial bleed with a lancet (Feather; 2,017-01). Serum was separated from blood using microvettes (Sarstedt; 16.443.100) and human insulin quantified using the Human Ultrasensitive Insulin ELISA.

A subset of mice were treated with alloxan to destroy endogenous mouse β-cells 12–16 weeks after transplantation. Alloxan (90 mg kg^−1^) was delivered by tail vein injection^21^. Loss of endogenous mouse β-cells was confirmed by measuring serum mouse C-peptide using a Mouse C-peptide ELISA (ALPCO Diagnostics; 80-CPTMS-E01). Blood glucose levels was periodically measured for mice fasted 16 h and for mice injected with 2 g kg^−1^ glucose.

### Global gene expression analysis

Clusters from several independent batches made from a subset of cell lines were dispersed with TrypLE, fixed in 4% PFA supplemented with RNasin (VWR; PAN2615) for 30 min on ice, stained with NKX6-1 and INS primary antibody in RNasin-containing buffer for 30 min on ice and stained with appropriate Alexa Fluor-488 and -647 secondary antibodies diluted in RNasin-containing buffer for 30 min on ice. INS+/NKX6-1+ cells were sorted using an FACSAria (BD Biosciences) and RNA extracted by first incubation sorted cells in digestion buffer (RecoverAll Total Nucleic Acid Isolation Kit; Ambion; AM1975) at 50 °C for 3 h then by following the instructions from the manufacturer. cDNA and cRNA was generated with Illumina TotalPrep RNA Amplifcation Kit (Life Technologies; AMIL1791). cRNA was hybridized to Human HT-12 Expression BeadChips (Illumina) with the Whole-Genome Expression Direct Hybridization kit (Illumina) and chips read with Illumina Beadstation 500. Data were analysed in GenomeStudio (Illumina) with background subtraction and rank-invariant normalization. Previous published data for HUES8 SC-β cells, undifferentiated HUES8, fetal β-cells and adult β-cells was also included in data analysis^17^,^22^. Hierarchical clustering was performed using Pearson's correlation and Ward linkage.

### Chemically induced stress

Cell clusters were dispersed by TrypLE Express treatment and plated onto growth factor reduced Matrigel-coated 96-well plates overnight in S6 media supplemented with 10 μM Alk5i, 1 μM T3 and 10 μM Y27632. Plated cells were then washed twice with S6 then cultured with S6 media alone or S6 media with combinations of chemical stressors (Supplementary Table 8) for up to 48 h. In a subset of experiments, cells were also cultured with 10 μM Alk5i. After chemical treatment, cells were fixed with 4% PFA for 30 min at RT, blocked with staining buffer for 30 min at RT, stained with primary antibody diluted in staining buffer overnight at 4 °C, stained with appropriate Alexa Fluor-488 and -594 secondary antibody diluted in staining buffer for 2 h at RT and stained with 4,6-diamidino-2-phenylindole. Stained cells were quantified by automated cell counting using Cellomics ArrayScanVTI, counting at least 1,000 cells per batch.

### Statistical analysis

Statistical significance was determined by the use of two-sided unpaired and paired *t*-tests, as appropriate. Data shown as mean±s.e.m. *n* indicates the total number of batches (biological replicates) represented by the data. The box and whisker plot was generated with defaulting statistical settings with BoxPlorR (http://boxplot.tyerslab.com/).

## Additional information

**Accession codes:** Global Gene Expression Analysis has been deposited in the NCBI Gene Expression Omnibus database under accession code GSE70901.

**How to cite this article:** Millman, J. R. *et al*. Generation of stem cell-derived β-cells from patients with type 1 diabetes. *Nat. Commun.* 7:11463 doi: 10.1038/ncomms11463 (2016).

## Supplementary Material

Supplementary Information Supplementary Figures 1-7 and Supplementary Tables 1-8

Supplementary Data 1 Microarray Data Set
