# Corrigendum: Generation of stem cell-derived β-cells from patients with type 1 diabetes

**DOI:** 10.1038/ncomms12379

**Published:** 2016-08-04

**Authors:** Jeffrey R. Millman, Chunhui Xie, Alana Van Dervort, Mads Gürtler, Felicia W. Pagliuca, Douglas A. Melton

Nature Communications 7: Article number: 1146310.1038/ncomms11463 (2016); Published: 05 10 2016; Updated: 08 04 2016.

The source of reagents for this Article was not fully acknowledged. The Acknowledgements should have included the following:

‘We thank Professors Robin Goland and Dieter Egli (https://www.cellhub.org) for patient fibroblasts.'

