# Supplementary material for: Generation of stem cell-derived β-cells from patients with type 1 diabetes
Source: Nat Commun. 2016 May 10;7:11463. doi: 10.1038/ncomms11463 (PMC4866045; doi:10.1038/ncomms11463)
Supplement: Supplementary Information — Supplementary Figures 1-7 and Supplementary Tables 1-8 [file ncomms11463-s1.pdf]

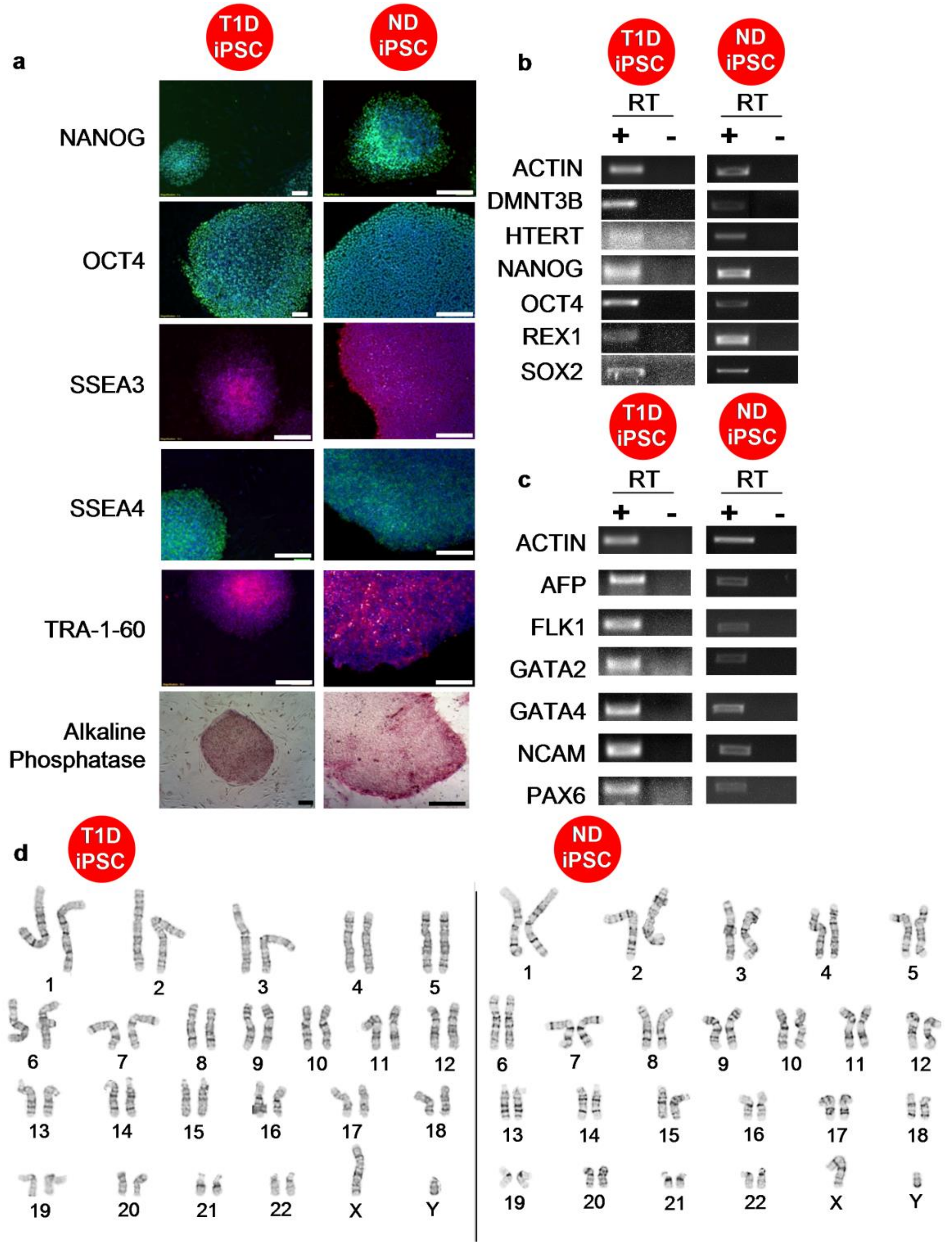

Supplementary Figure 1: T1D hiPSC express pluripotency markers, show differentiation potential in an embryoid body differentiation assay, and are karyotypically normal. (**a**) Undifferentiated T1D (T1D-1) and ND (ND-2) hiPSC stained for NANOG (green), OCT4 (green), SSEA3 (red), SSEA4 (green), or TRA-1-60

(red) with DAPI or separately with Alkaline Phosphatase. Scale bar=100 µm. (**b**) Gene expression of ACTIN, DMNT3B, HTERT, NANOG, OCT4, REX1, and SOX2 in undifferentiated hiPSC. (**c**) Gene expression of ACTIN, AFP, FLK1, GATA2, GATA4, NCAM, and PAX6 for hiPSC differentiated in embryoid bodies. (**d**) Images of karyotype analysis.

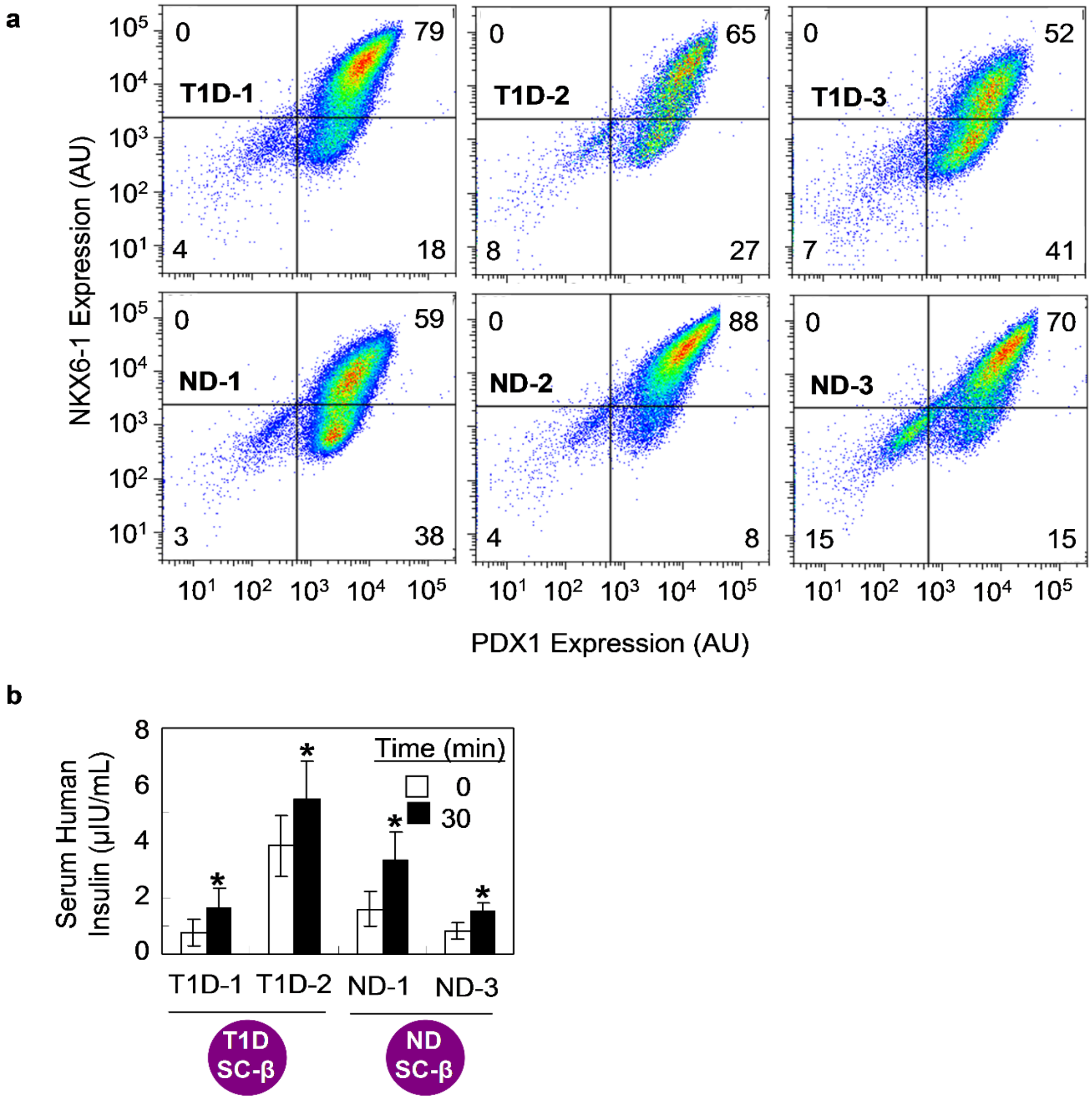

Supplementary Figure 2: T1D hiPSC can differentiate to pancreatic progenitors that can spontaneously undergo in vivo maturation to glucose-responsive cells. (**a**) Flow cytometry plots of T1D and hiPSC differentiated with pancreatic progenitor planar protocol and stained for NKX6-1 and PDX1. (**b**) In vivo glucose-stimulated insulin secretion of mice transplanted 16 wk prior with a subset of hiPSC lines differentiated with pancreatic progenitor planar protocol. n=4, 5, 5, 7 for T1D-1, T1D-2, ND-1, and ND-3, respectively. *$p<0.05$ comparing 30 min to 0 min (two-sided paired t-test). Data shown as mean±SEM.

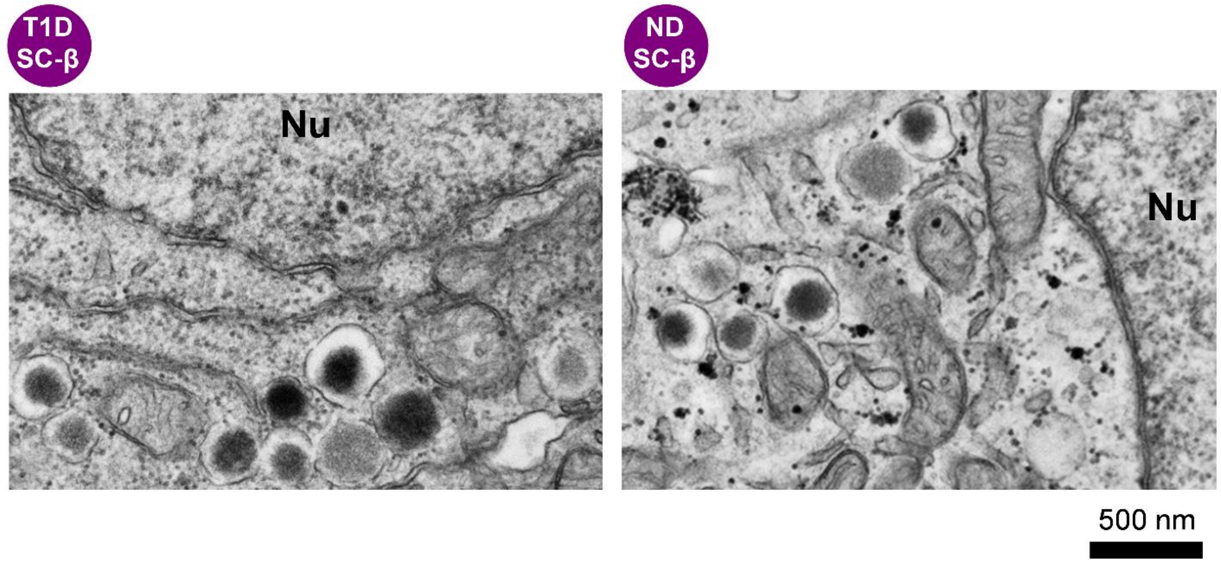

Supplementary Figure 3: Electron microscopy of sectioned cells showing insulin granules in T1D (left; T1D-2) and ND (right; ND-2) SC-β cells. Samples taken after 10-17 days in Stage 6.

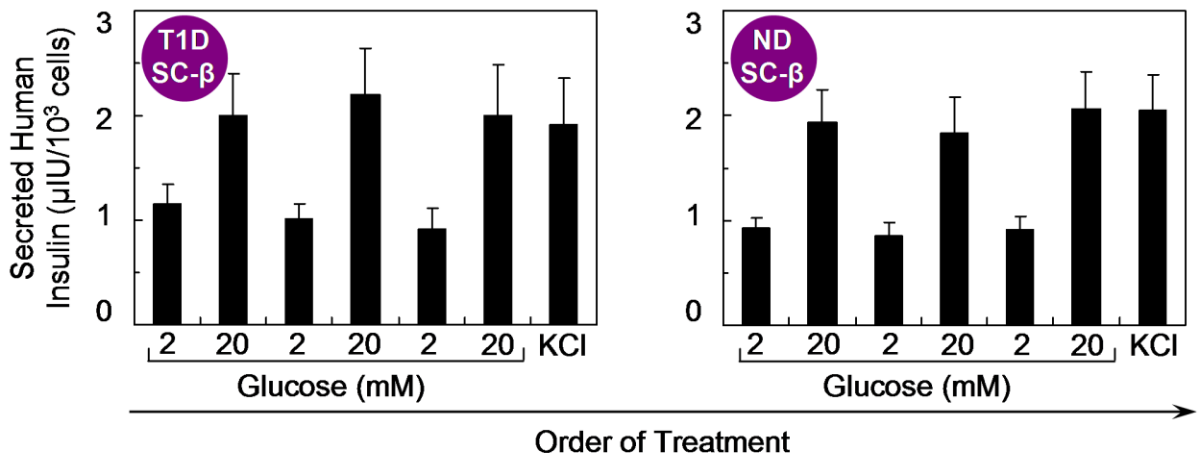

Supplementary Figure 4: T1D and ND SC-β cells respond to 3 sequential glucose challenges and treatment with 30 mM KCl. Average ELISA measurements of secreted human insulin. n=9 and 9 SC-β cells batches each consisting of 3 T1D donors and 3 ND donor. Data shown as mean±SEM. Samples taken after 10-17 days in Stage 6.

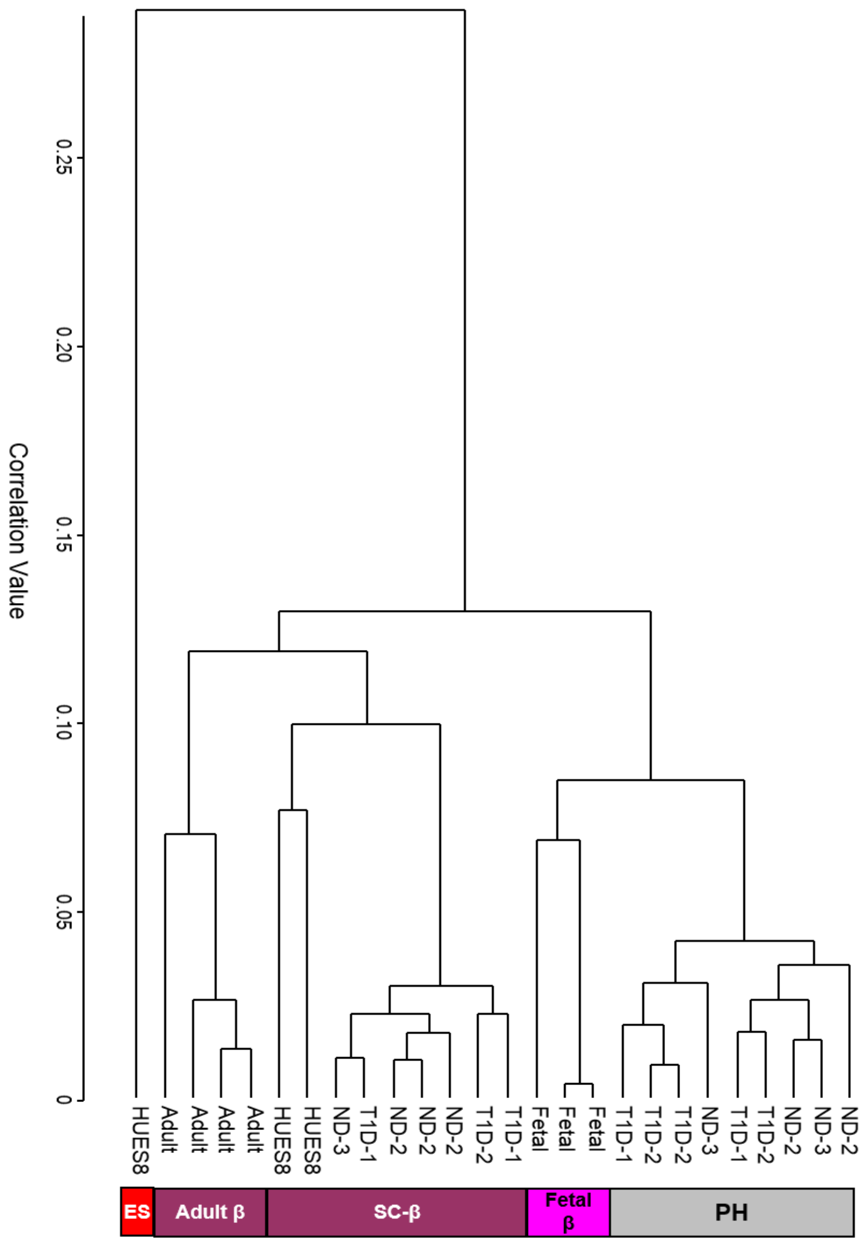

Supplementary Figure 5: Hierarchal clustering based on global gene expression of undifferentiated cells (HUES8), PH cells (ND-2, ND-3, T1D-1, T1D2), Fetal β cells, SC-β cells (HUES8, ND-2, ND-3, T1D-1, T1D-2), and Adult β cells. Data for undifferentiated cells (HUES8), Fetal β cells, and Adult β cells come from Hrvatin et al.

2014 and SC-β cells (HUES8) from Pagliuca et al. 2014. The PH cells are dysfunctional insulin-expressing cells that are often polyhormonal (PH) and were generated with a control protocol that does not produce SC-β cells. Samples taken after 10-17 days in Stage 6.

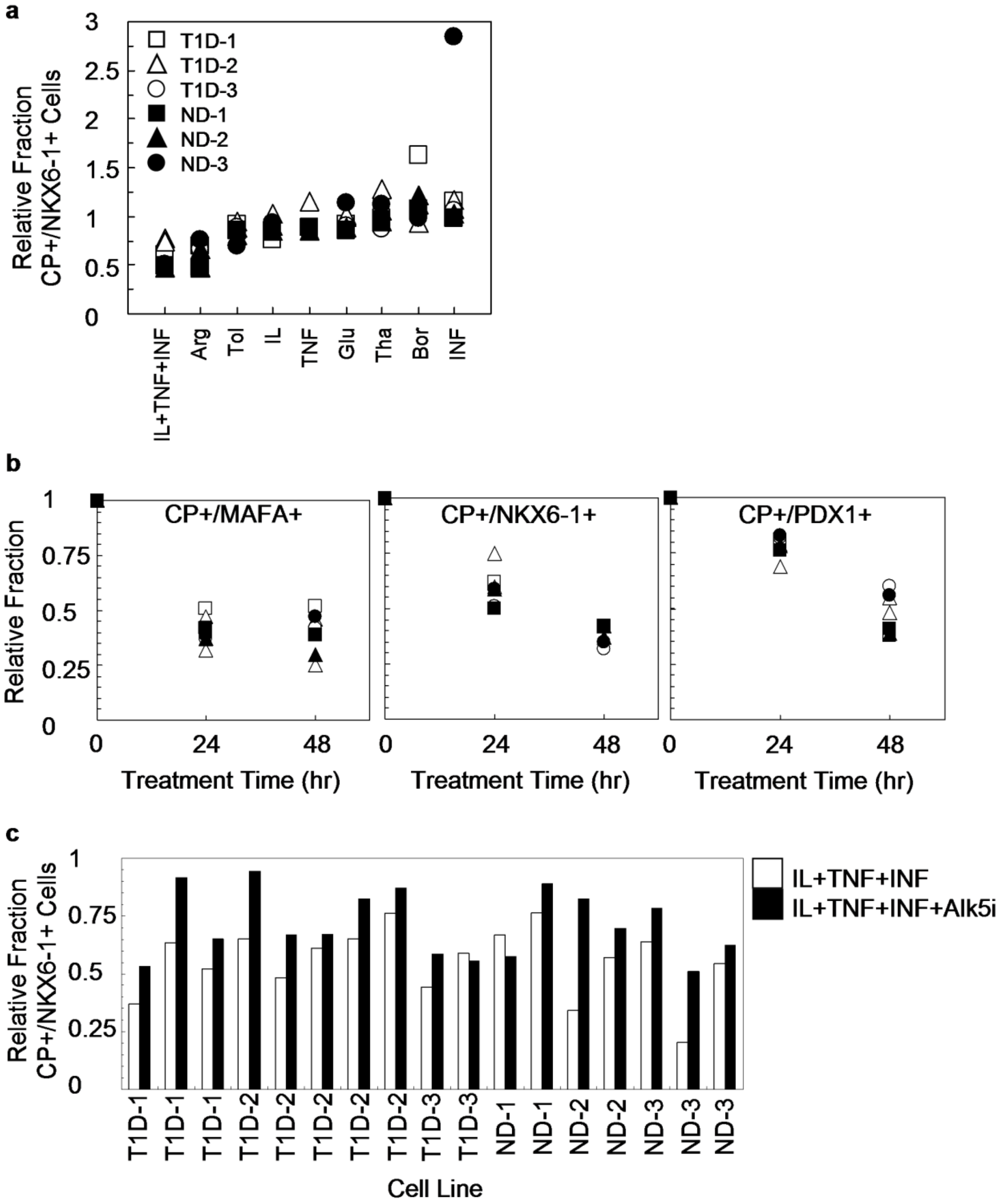

Supplementary Figure 6: Individual measurements that make up Fig. 3b, 3c, and 3f. (**a**) The relative fraction of T1D and ND SC-β cells immunostained for C-peptide (CP) and NKX6-1 treated 24 h with the indicated stressor normalized by untreated cells.

n=3 T1D and 4 ND SC-β cells batches. (**b**) The relative fraction of cells immunostained for C-peptide and PDX1, NKX6-1, or MAFA treated up to 48 h with IL-1β, TNF-α, and INF-γ n=3 T1D and 4 ND SC-β cells batches. (**c**) Relative fraction of C-peptide+/NKX6-1+ cells treated with IL-1β, TNF-α, and INF-γ either without (left) or with (right) Alk5i for 24 h. n=10 T1D and 7 ND SC-β cells batches (17 batches total). Quantification of immunostained cells in this figure was performed with Cellomics ArrayScanVTI. Samples taken after 10-17 days in Stage 6. IL, IL-1β; TNF, TNF-α; INF, INF-γ; Arg, Arginine; Tol, Tolbutamide; Glu, Glucose; Tha, Thapsagargin; Bor, Bortezomib.

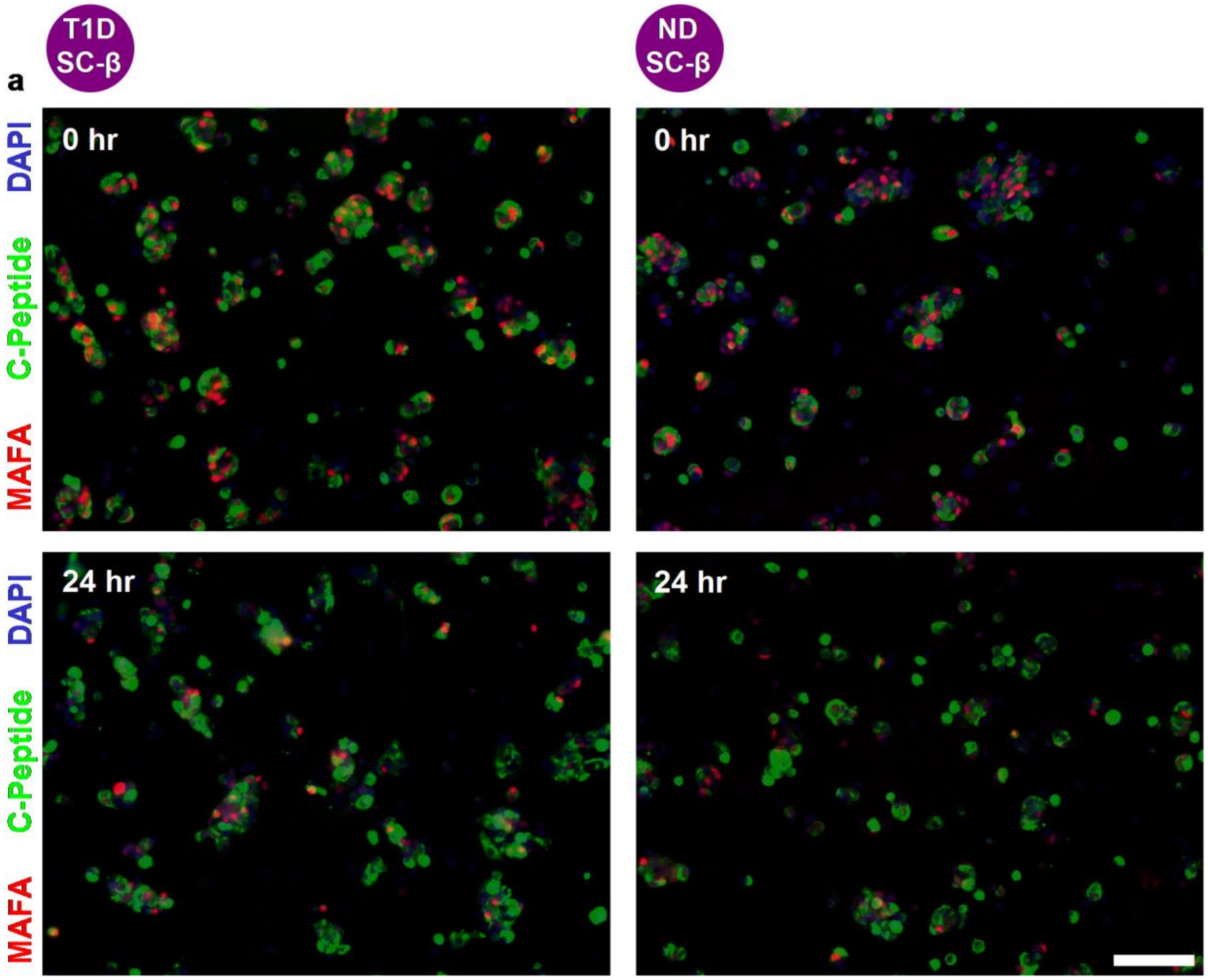

Supplementary Figure 7: MAFA expression of dispersed and plated T1D and ND SC-β cells. (**a**) Cells before treatment with IL+TNF+INF. (**b**) Cells 24 hr after treatment with IL+TNF+INF. Scale bar=100 µm. Samples taken after 10-17 days in Stage 6.

Supplementary Table 1: ELISA measurements of human insulin in the serum of mice transplanted with T1D and ND SC-β cells 2 wk prior.

				Human Insulin (µIU/mL)	
Cell Type	Cell Line	Batch#	ms#	0'	30'
T1D	T1D-1	1	1	4.43	5.44
	T1D-1	1	2	8.27	10.85
	T1D-1	1	3	10.10	22.28
	T1D-1	2	4	13.71	13.43
	T1D-1	2	5	13.96	19.03
	T1D-1	2	6	18.69	21.33
	T1D-2	3	7	1.45	4.85
	T1D-2	3	8	0.62	0.95
	T1D-2	3	9	1.46	2.50
	T1D-2	3	10	1.09	2.27
	T1D-2	3	11	1.09	6.13
	T1D-2	4	12	3.93	6.64
	T1D-2	4	13	4.75	5.69
	T1D-2	4	14	1.86	5.69
	T1D-2	4	15	4.69	5.19
	T1D-2	5	16	1.75	3.06
	T1D-2	5	17	1.44	1.18
	T1D-2	5	18	0.60	0.51
	T1D-2	5	19	0.76	1.97
	T1D-3	6	20	3.55	5.94
	T1D-3	6	21	3.24	4.75
	T1D-3	6	22	8.90	9.40
	T1D-3	6	23	3.11	6.20
	T1D-3	6	24	3.81	3.62
	T1D-3	6	25	2.67	3.18
	T1D-3	7	26	2.61	3.68
	T1D-3	7	27	5.19	6.89
	T1D-3	7	28	3.18	2.55
	T1D-3	7	29	6.01	6.51
	T1D-3	7	30	1.23	3.24
	T1D-3	7	31	1.42	1.04
	T1D-3	7	32	3.87	0.41
ND	ND-1	8	33	5.19	7.06
	ND-1	8	34	5.74	5.72
	ND-1	8	35	5.19	12.36

ND-1	8	36	4.32	5.68
ND-1	8	37	3.87	3.47
ND-1	8	38	5.16	9.41
ND-1	8	39	5.06	5.31
ND-1	8	40	7.44	24.72
ND-1	8	41	8.09	14.08
ND-1	9	42	1.68	2.93
ND-1	9	43	1.63	2.48
ND-1	9	44	1.68	2.38
ND-1	9	45	1.92	2.46
ND-1	10	46	2.36	4.24
ND-1	10	47	0.47	1.25
ND-1	10	48	0.87	3.29
ND-1	10	49	1.05	3.82
ND-1	10	50	1.10	1.01
ND-2	11	51	4.81	6.38
ND-2	11	52	8.58	13.43
ND-2	11	53	3.05	0.85
ND-2	11	54	1.10	10.47
ND-2	11	55	5.57	11.54
ND-2	11	56	5.63	5.63
ND-2	12	57	24.06	12.87
ND-2	12	58	10.07	16.96
ND-2	12	59	7.67	7.26
ND-2	12	60	10.20	12.71
ND-2	13	61	6.02	6.03
ND-2	13	62	1.33	2.45
ND-2	13	63	2.39	6.83
ND-2	13	64	2.81	3.30
ND-2	13	65	2.23	2.37
ND-2	14	66	4.12	2.23
ND-2	14	67	2.67	4.81
ND-2	14	68	9.34	6.32
ND-2	14	69	1.54	6.89
ND-2	14	70	7.89	11.92
ND-2	14	71	5.57	3.93
ND-3	15	72	12.92	11.73
ND-3	15	73	4.62	5.75
ND-3	15	74	6.70	5.13
ND-3	15	75	2.86	5.31
ND-3	15	76	6.20	9.28
ND-3	15	77	3.68	8.90
ND-3	16	78	1.64	2.08

ND-3	16	79	2.98	36.75
ND-3	16	80	2.68	4.35

Supplementary Table 2: Media formulations for SC-β cell differentiation.

Stage	Day	Basal Media	Factor	Concentration	Company	Part Number
**Stage 1**	Day 1	S1	Activin A<br>CHIR99021	100 ng/mL<br>14 μg/mL	R&D Systems<br>Stemgent	338-AC<br>04-0004-10
	Day 2	S1	Activin A<br>CHIR99021	100 ng/mL<br>None	R&D Systems	338-AC
	Day 3		No Feed			
**Stage 2**	Day 1	S2	KGF	50 ng/mL	Peprotech	AF-100-19
	Day 2		No Feed			
	Day 3	S2	KGF	50 ng/mL	Peprotech	AF-100-19
**Stage 3**	Day 1	S3	LDN193189<br>KGF<br>SANT1<br>PdBU<br>Y27632<br>RA	200nM<br>50 ng/mL<br>0.25 μM<br>500 nM<br>10 μM<br>2 μM	Thermo Fisher Scientific<br>Peprotech<br>Sigma Aldrich<br>EMD Millipore<br>Abcam<br>Sigma Aldrich	NC0054000<br>AF-100-19<br>S4572<br>524390<br>ab120129<br>R2625
	Day 2	S3	LDN193189<br>KGF<br>SANT1<br>PdBU<br>Y27632<br>RA	None<br>50 ng/mL<br>0.25 μM<br>500 nM<br>10 μM<br>2 μM	 Peprotech<br>Sigma Aldrich<br>EMD Millipore<br>Abcam<br>Sigma Aldrich	 AF-100-19<br>S4572<br>524390<br>ab120129<br>R2625
**Stage 4**	Day 1	S3	KGF<br>SANT1<br>Y27632<br>Activin A<br>RA	50 ng/mL<br>0.25 μM<br>10 μM<br>5 ng/mL<br>0.1 μM	Peprotech<br>Sigma Aldrich<br>Abcam<br>R&D Systems<br>Sigma Aldrich	AF-100-19<br>S4572<br>ab120129<br>338-AC<br>R2625
	Day 2		No Feed			
	Day 3	S3	KGF<br>SANT1<br>Y27632<br>Activin A<br>RA	50 ng/mL<br>0.25 μM<br>10 μM<br>5 ng/mL<br>0.1 μM	Peprotech<br>Sigma Aldrich<br>Abcam<br>R&D Systems<br>Sigma Aldrich	AF-100-19<br>S4572<br>ab120129<br>338-AC<br>R2625
	Day 4		No Feed			
	Day 5	S3	KGF<br>SANT1<br>Y27632<br>Activin A<br>RA	50 ng/mL<br>0.25 μM<br>10 μM<br>5 ng/mL<br>0.1 μM	Peprotech<br>Sigma Aldrich<br>Abcam<br>R&D Systems<br>Sigma Aldrich	AF-100-19<br>S4572<br>ab120129<br>338-AC<br>R2625
**Stage 5**	Day 1	BE5	SANT1<br>RA<br>XXI<br>Alk5i	0.25 μM<br>0.1 μM<br>1 μM<br>10 μM	Sigma Aldrich<br>Sigma Aldrich<br>EMD Millipore<br>Axxora	S4572<br>R2625<br>595790<br>ALX-270-445-M005

			T3<br>Betacellulin	1 µM<br>20 ng/mL	EMD Biosciences<br>R&D Systems	64245<br>261-CE-050
	Day 2		No Feed			
	Day 3		SANT1<br>RA<br>XXI<br>Alk5i<br>T3<br>Betacellulin	0.25 µM<br>0.1 µM<br>1 µM<br>10 µM<br>1 µM<br>20 ng/mL	Sigma Aldrich<br>Sigma Aldrich<br>EMD Millipore<br>Axxora<br>EMD Biosciences<br>R&D Systems	S4572<br>R2625<br>595790<br>ALX-270-445-M005<br>64245<br>261-CE-050
	Day 4		No Feed			
	Day 5		SANT1<br>RA<br>XXI<br>Alk5i<br>T3<br>Betacellulin	None<br>25 nM<br>1 µM<br>10 µM<br>1 µM<br>20 ng/mL	Sigma Aldrich<br>Sigma Aldrich<br>EMD Millipore<br>Axxora<br>EMD Biosciences<br>R&D Systems	S4572<br>R2625<br>595790<br>ALX-270-445-M005<br>64245<br>261-CE-050
	Day 6		No feed			
	Day 7		SANT1<br>RA<br>XXI<br>Alk5i<br>T3<br>Betacellulin	None<br>25 nM<br>1 µM<br>10 µM<br>1 µM<br>20 ng/mL	Sigma Aldrich<br>Sigma Aldrich<br>EMD Millipore<br>Axxora<br>EMD Biosciences<br>R&D Systems	S4572<br>R2625<br>595790<br>ALX-270-445-M005<br>64245<br>261-CE-050
**Stage 6**	EOD	S6	Alk5i<br>T3	10 µM<br>1 µM	Axxora<br>EMD Biosciences	ALX-270-445-M005<br>64245

EOD: Feed every other day

Supplementary Table 3: The basal media formulations for S1, S2, S3, and S5.

	S1	S2	S3	S5	Company	Part Number
MCDB131 (L)	1	1	1	1	Cellgro	15-100-CV
Glucose (g)	0.44	0.44	0.44	3.6	Sigma	G7528
NaHCO3 (g)	2.46	1.23	1.23	1.754	Sigma	S3817
FAF-BSA (g)	20	20	20	20	Proliant	68700
ITS-X (mL)	0.02	0.02	5	5	Invitrogen	51500056
Glutamax (mL)	10	10	10	10	Invitrogen	35050079
Vitamin C (mg)	44	44	44	44	Sigma	A4544
Heparin (mg)	0	0	0	10	Sigma	H3149
P/S (mL)	10	10	10	10	Mediatech	30-002-CI

Supplementary Table 4: Media formulations for PDX1+/NKX6-1+ pancreatic progenitors planar differentiation.

Stage	Days	Basal media	Factors	Concentration
Stage 1	1	S1	Activin A<br>CHIR99021	100 ng/mL<br>14 µg/mL
	3	S1	Activin A	100 ng/mL
Stage 2	2	S2	KGF	25 ng/mL
Stage 3	2	S3	KGF<br>PdBU<br>SANT1<br>RA<br>LDN	25 ng/mL<br>500 nM<br>0.25 µM<br>2 µM<br>100 nM
Stage 4	3	S3	PdBU<br>SANT1<br>RA<br>LDN	500 nM<br>0.25 µM<br>0.1 µM<br>100 nM

Feed every day

Supplementary Table 5: Media formulations for PH cell planar differentiation.

Stage	Days	Basal media	Factors	Concentration
Stage 1	1	S1	Activin A	100 ng/mL
			CHIR99021	14 µg/mL
	3	S1	Activin A	100 ng/mL
Stage 2	2	S2	KGF	25 ng/mL
Stage 3	2	S3	KGF	25 ng/mL
			PdBU	500 nM
			SANT1	0.25 µM
			RA	2 µM
			LDN	100 nM
Stage 4	7	S3	Alk5i	10 µM
			XXI	1 µM

Feed every day

Supplementary Table 6: Primary antibodies used in study.

Target	Species	Company	Part Number	Dilution
PDX1	Goat	R&D Systems	AF2419	1:300
NKX6-1	Mouse	University of Iowa Developmental Hybridoma Bank	F55A12-supernatant	1:100
MAFA	Rabbit	Abcam	Ab26405	1:300
C-peptide	Rat	University of Iowa Developmental Hybridoma Bank	GN-ID4	1:300
GCG	Mouse	Abcam	ab82270	1:300
Insulin	Guinea pig	Dako	A0564	1:300

Supplementary Table 7: Formulation of krebs buffer.

Volume		Component
1000.0	mL	Deionized Water
25.6	mL	NaCl [5M]
2.5	mL	KCl [2M]
2.7	mL	CaCl2 [1M]
1.2	mL	MgSO4 [1M]
10.0	mL	Na2HPO4 [0.1M]
1.2	mL	KH2PO4 [1M]
5.0	mL	NaHCO3 [1M]
10.0	mL	HEPES [1M]
1.0	g	BSA

Supplementary Table 8: Chemical stressors used in study.

Compound	Concentration	Company	Part Number
IL-1β	0.1 μg/mL	R&D Systems	201-LB-005
TNF-α	0.5 μg/mL	R&D Systems	210-TA-010
INF-γ	3.6 μg/mL	R&D Systems	285-IF-100
Arginine	10 mM	Sigma	A500G
Tolbutamide	250 μM	Sigma	T0891
Glucose	25 mM	Sigma	G7528
Thapsagargin	1 μM	EMD Chemicals	586005
Bortezomib	30 nM	Santa Cruz Biotechnology	sc-217785
